# Division of Communicable Diseases

**Published:** 1916-10

**Authors:** Hermann M. Biggs

**Affiliations:** Commissioner of Health. New York State Department of Health, Albany


### CORRESPONDENCE.

This department is intended for the presentation of news and views not coming within the scope of other departments, and particularly to afford opportunity for discussion and criticism of views elsewhere expressed.

---

**Division of Communicable Diseases**
New York State Department of Health, Albany.
September 10, 1916.

Deaths in New York State 1915—Diphtheria 1751; Typhoid Fever 748; Measles 834; Whooping Cough 741; Scarlet Fever 413.

Dear Doctor:

Reports of cases during last month indicate that **typhoid fever** is unusually prevalent in your county. In order to control this disease the Department offers the following suggestions:

1. Report the case immediately to the health officer. Advise him regarding source of water and milk and any other possible source of infection.

2. In suspicious cases obtain a blood culture, if possible. After the 9th day send a specimen of blood to the laboratory for Widal test. The health officer has Widal outfits.

3. Use the same care in disinfecting the excreta of a **suspicious case of typhoid fever** that is used for a true case.

4. A disinfecting solution should always be kept on hand, made by placing ½-lb. of chloride of lime in one gallon of water. Discharges from the bowels and urine should be received in bed-pans or other vessels containing a small amount of disinfectant. Later the excreta should be liberally covered with the disinfectant and allowed to stand for one hour; then the receptacle with its contents may be emptied into a water-closet or trench. The trench should be located a considerable distance from water supplies and should be 1 ft. wide, 3 ft. deep and 4 ft. long and covered with a plank to exclude snow or flies.

The hands of the nurse or attendant should be thoroughly washed with soap and water and disinfected with a 1 to 2000 bichloride solution immediately after caring for the bed-pan, after handling the patient, and before meals.

5. Other members of the household, if they have not had typhoid fever and are under 50 years of age, should be immunized with typhoid vaccine. This vaccine may be obtained from the health officer.

6. Other members of the family may pursue their usual occupations except as the health officer may otherwise direct.

7. If the patient lives on a dairy farm or at a factory or store where dairy products are sold regulation 37, Chapter II of the Sanitary Code must be observed.

8. Boil all drinking water and pasteurize the milk until the source of the infection is otherwise determined.

9. See that the Department's circulars on this disease have been distributed by the health officer to the homes of infected cases.

Very truly yours,
HERMANN M. BIGGS,
Commissioner of Health.

